# Simultaneously genetic selection of wheat yield and grain protein quality in rice–wheat and soybean–wheat cropping systems through critical nitrogen efficiency-related traits

**DOI:** 10.3389/fpls.2022.899387

**Published:** 2022-09-29

**Authors:** Yufeng Chen, Kun Wang, Haolan Chen, Hongkun Yang, Ting Zheng, Xiulan Huang, Gaoqiong Fan

**Affiliations:** ^1^Crop Ecophysiology and Cultivation Key Laboratory of Sichuan Province, Sichuan Agricultural University, Chengdu, China; ^2^State Key Laboratory of Crop Gene Exploration and Utilization in Southwest China, Ministry of Science and Technology, Sichuan Agricultural University, Chengdu, China; ^3^Key Laboratory of Crop Eco-Physiology and Farming System in Southwest China, Ministry of Agriculture and Rural Affairs, Sichuan Agricultural University, Chengdu, China

**Keywords:** grain yield, grain protein content (GPC), gluten index, nitrogen use efficiency (NUE), genetic selection

## Abstract

Analyzing the contribution of nitrogen (N) uptake and its utilization in grain yield and protein quality-related traits in rice-wheat (RW) and soybean-wheat (SW) cropping systems is essential for simultaneous improvements in the two target traits. A field experiment with nine wheat genotypes was conducted in 2018–19 and 2019–20 cropping years to investigate N uptake and utilization-related traits associated with high wheat yield and good protein quality. Results showed that N uptake efficiency (NUpE) in the RW cropping system and N utilization efficiency (NUtE) in the SW cropping system explained 77.6 and 65.2% of yield variation, respectively, due to the contribution of fertile spikes and grain number per spike to grain yield varied depending on soil water and N availability in the two rotation systems. Lower grain protein content in the RW cropping system in comparison to the SW cropping system was mainly related to lower individual N accumulation at maturity, resulting from higher fertile spikes, rather than N harvest index (NHI). However, NHI in the SW cropping system accounted for greater variation in grain protein content. Both gluten index and post-anthesis N uptake were mainly affected by genotype, and low gluten index caused by high post-anthesis N uptake may be related to the simultaneous increase in kernel weight. N remobilization process associated with gluten quality was driven by increased sink N demand resulting from high grain number per unit area in the RW cropping system; confinement of low sink N demand and source capability resulted in low grain number per spike and water deficit limiting photosynthesis of flag leaf in the SW cropping system. CY-25 obtained high yield and wet gluten content at the expense of gluten index in the two wheat cropping systems, due to low plant height and high post-anthesis N uptake and kernel weight. From these results, we concluded that plant height, kernel weight, and post-anthesis N uptake were the critically agronomic and NUE-related traits for simultaneous selection of grain yield and protein quality. Our research results provided useful guidelines for improving both grain yield and protein quality by identifying desirable N-efficient genotypes in the two rotation systems.

## Introduction

Rice-wheat (RW) and soybean-wheat (SW) cropping systems are two dominant crop rotation systems in the world (Hu et al., 2019; Lian et al., 2020). The previous crop affects subsequent wheat growth and development (Singh et al., 2008), resulting in different wheat yield and grain protein quality, which can be explained by the effects of water, nitrogen (N), and their effect on N uptake and N utilization (Vashisht et al., 2021). Wheat cultivated from the RW cropping system is often used for wine-making (Peng Q. et al., 2022) and biscuit processing due to its low grain protein content, while wheat cultivated from the SW cropping system is processed into bread, steamed bread, and noodles due to its relatively high grain protein content (Ma et al., 2019). However, simultaneous selection of grain yield and grain protein quality is challenging due to the negative correlation between grain yield and grain N content (Rapp et al., 2018) and the complex effects of genotype, environment, and their effect on grain yield and protein quality-related traits (Saleem et al., 2015). Thus, identifying high-yielding wheat with diverse processing requirements through genetic selection for critical N use efficiency (NUE)- related traits may help break the negative relationship between grain yield and grain N content.

Nitrogen is an essential component of chlorophyll, photosynthetic enzymes, and storage proteins (Luo et al., 2021), and it is also linked to grain yield and protein quality. Although genetic improvement of NUE is known to increase the grain yield (Yang et al., 2021), the relative contribution of N uptake efficiency (NUpE) and N utilization efficiency (NUtE) to yield, and the effects of NUE-related traits on the improvements of yield, gluten content, and gluten index in the two rotation systems remain unclear. Moreover, critical NUE-related traits associated with simultaneous selection for high yield and target grain protein quality in the two rotation systems warrant further exploration.

Nitrogen uptake efficiency represents the capability of the root system to take up N from soil (Bryant Schlobohm et al., 2020), and a higher NUpE entails more fertile tillers and (or) higher individual N accumulation on maturity. Improving N acquisition by identifying N uptake-efficient genotypes (Chandna et al., 2012) is economical in both high- and low-input cropping systems. This is because of the fact that high N fertilizer input reduces N recovery efficiency, thereby increasing the risk of environmental pollution (Zhang et al., 2021). However, the effect of soil water and N supply on the genetic selection of N efficient traits should be considered due to the interaction between genotype and environment (Lemaire and Ciampitti, 2020). The soil water, N reserves, and enzyme activities in the RW cropping system were significantly higher than that in the SW system (Sharma et al., 2021); while in the SW cropping system, soil water restricts the root N uptake, thus presenting lower NUpE and grain yield in comparison to the RW cropping system (Gao et al., 2009). Although improving the NUpE is beneficial to increase grain yield, the relative contribution of fertile tillers and individual N accumulation at maturity to NUpE and the effects of NUpE-related traits on grain yield and protein quality-related traits remains unknown.

Nitrogen utilization efficiency represents the capability of crops to convert the absorbed N into grain yield (De Oliveira Silva et al., 2020), and high NUtE requires plants to maximize post-anthesis N uptake, N remobilization efficiency, and N harvest index (NHI) (Kichey et al., 2007). In the RW cropping system, increased post-anthesis N uptake resulting from improved soil water and N environments (Li et al., 2019) improves the NHI and yield by increasing post-anthesis photosynthesis but decreasing the N remobilization efficiency (Wu et al., 2018), while in the SW cropping system, soil water deficit suppresses stomatal conductance in leaves, decreases photosynthesis, accelerates leaf senescence, resulting in decreased plant biomass and grain yield (Olszewski et al., 2008). However, the complex and controversial effects of post- anthesis N uptake and N remobilization on grain protein quality have also been reported (Barraclough et al., 2014; Ding et al., 2016). In this context, the relative importance of NUtE-related traits in the simultaneous selection for high yield and target protein quality in the two wheat cropping systems warrants further research.

There is a universal and negative correlation between grain yield and grain protein content (Lollato et al., 2019; Crosta et al., 2021); hence, this increase in grain protein content was considered at the expense of grain yield (Pompa et al., 2011). However, this negative correlation was affected by soil water and N environment (Dupont et al., 2006; Triboi et al., 2006). Previous studies on grain protein deviation have identified genotypes with both high grain yield and protein content, attributed to improvements of post-anthesis N uptake or N remobilization efficiency (Bogard et al., 2010; Latshaw et al., 2016; Thorwarth et al., 2018). However, the positive and negative effects of post-anthesis N uptake and N remobilization efficiency on grain yield and protein quality have also been reported (Oosterom et al., 2010; Barraclough et al., 2014; Kong et al., 2015). Hence, the complexity of yield and grain protein quality implies that a single NUE-related trait cannot explain simultaneous improvements in grain yield and protein quality, due to the effects of genotype and environment as well as their interaction on these traits. The associations between NUE and yield, and grain protein content have been explored previously (Kayan et al., 2020; Ma et al., 2021). Our previous results showed that genetic gains in grain yield and NUE in a historical set of cultivars were associated with improvements in light interception, solar energy conversion, and biomass partitioning efficiency (Yang et al., 2021). However, comparative studies on the contribution of N uptake, partitioning, remobilization, and utilization in simultaneous selection for grain yield and target protein quality-related traits of different wheat genotypes in the two rotation systems are lacking. Therefore, the objectives of the current study were to (i) investigate the relative contributions of NUpE and NUtE to genetic variation in grain yield and the effects of NUE-related traits on grain protein quality-related traits in the two rotation systems; (ii) and to identify crucially agronomic and NUE-related traits contributing to simultaneous selection for wheat yield and target protein quality in the two rotation systems.

## Materials and methods

### Site and experiment description

**Figure 1:**
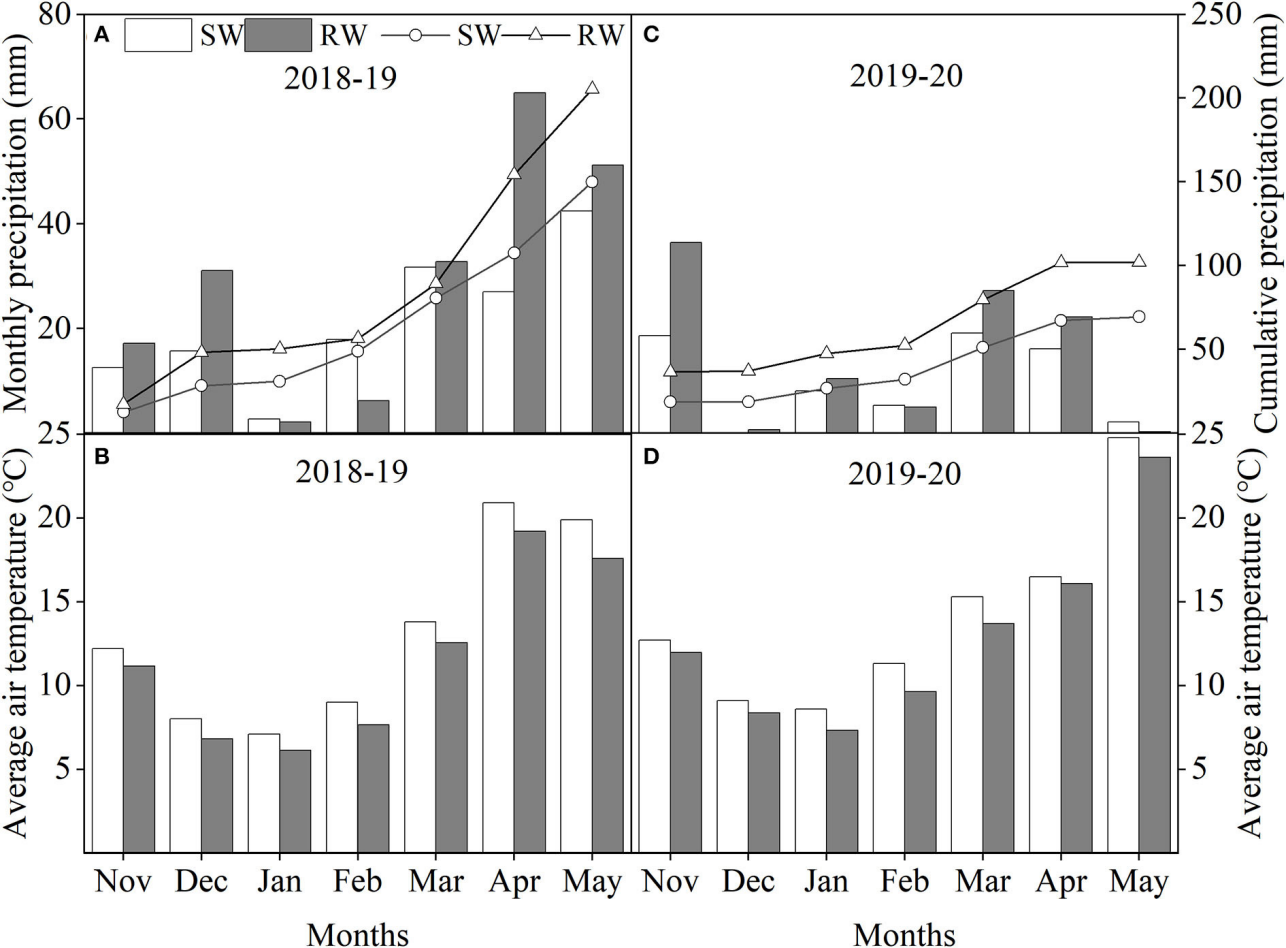
Monthly precipitation, cumulative precipitation during 2018–19 **(A)** and 2019–20 **(C)** cropping years and average air temperature during 2018–19 **(B)** and 2019–20 **(D)** cropping years in two wheat cropping systems. All data were collected from sowing to physiological maturity in each cropping year. SW, soybean-wheat cropping system; RW, rice-wheat cropping system.

Field trials were conducted during 2018–19 and 2019–20 cropping years at Renshou (29°51^′^N, 104°12^′^E) and Dayi (30°49^′^N, 103°58^′^E) experimental stations in southwestern China. SW and RW cropping systems were the dominant rotation systems in Renshou and Dayi experiment stations, respectively. According to the FAO classification (Faostat, 2016), soil in both SW and RW cropping systems were purple soil and clay loam, respectively. The respective average physicochemical properties soil (0–20 cm depth) before wheat sowing (Hawkesford, 2017) in SW and RW cropping systems were as follows: pH = 6.84 and 7.46; soil water storage = 320 mm and 470 mm; total N = 1.25 and 2.04 g kg^−1^; available N = 100.50 and 153.67 mg kg^−1^; available P = 4.73 and 23.51 mg kg^−1^; available K = 162.99 and 221.30 mg kg^−1^; and organic matter = 15.69 and 58.80 g kg^−1^. Monthly precipitation, cumulative precipitation, and average air temperature in two wheat cropping systems during 2018–19 and 2019–20 cropping years are shown in Figure 1. Cumulative precipitation of the SW cropping system were 149.9 mm and 69.3 mm in 2018–19 and 2019–20 cropping years, respectively, and the cumulative precipitation of the RW cropping system in 2018–19 and 2019–20 cropping years were 205.3 mm and 101.9 mm, respectively. Therefore, 2019–20 cropping year was considered a typical drought year.

A randomized complete block design with three replicates was conducted in both experimental stations. Each plot was 4 m long and 3 m wide. Based on our previous two-year field experiment, nine typical wheat genotypes, including landraces, advanced landraces and modern genotypes were selected from 32 wheat (*Triticum aestivum* L.) genotypes released in Southwest China between 1965 and 2017. All selected genotypes are registered and grown widely (>100,000 ha per year) in Southwest China. The selected genotypes presented noticeable genetic variations in grain yield, protein quality, and food use properties (Table 1). Dwarf genes and harvest index of nine wheat genotypes are also shown in Table 1. Wheat was sown at the end of October. Row spacing was maintained at 0.2 m to yield an initial seedling population of 180 seedlings m^−2^. Before sowing, 150 kg ha^−1^ N (at a 6:4 sowing: jointing ratio), 75 kg ha^−1^ P, and 75 kg ha^−1^ K were applied as urea, P_2_O_5_, and K_2_O, respectively. Commercial herbicides, pesticides, and fungicides were applied monthly after tillering to avoid yield loss. Other management practices followed the local practices.

**Table 1:** Introduction about the basic information of nine wheat genotypes.

**Genotype**		**Breeding** **groups**	**Grain** **protein** **content (%)**	**Wet gluten** **content (%)**	**Gluten index** **(%)**	**Grain yield** **(t ha^−1^)**	**Food** **useproperty**	**Dwarfing** **genes**	**Harvest** **index**
G_1_	SM-482	L	14.3	31.7	88	3.2	Noodle	*Rht* 8	0.46
G_2_	CM-39	AD	15.0	36.0	97	3.2	Noodle	*Rht* 8	0.39
G_3_	CM-66	AD	14.1	30.0	67	3.4	Biscuit	*Rht* 8	0.43
G_4_	NM-101	L	15.5	40.4	54	2.9	Noodle	*Rht* D1b	0.34
G_5_	MY-26	AD	13.9	37.3	73	3.3	Bread	*Rht* 8	0.46
G_6_	CM-81	L	13.5	30.3	84	3.1	Biscuit	*Rht* 8	0.47
G_7_	CY-25	S × AD	14.2	38.2	52	4.1	Noodle	*Rht* 8	0.47
G_8_	MM-51	AD	11.6	27.4	87	3.9	Biscuit	*Rht* D1b	0.47
G_9_	CM-104	S × AD	13.6	30.2	89	3.9	Noodle	*Rht* 8	0.49

G, genotype; L, landrace; AD, advanced landrace; S × AD, synthetic hexaploid wheat × advanced landrace.

All data were the mean values of nine wheat genotypes in 2016–17 and 2017–18 cropping years.

### Sampling methods and measurements

#### Yield, yield components, and yield-related traits

Numbers of initial seedling, maximum seedling, and fertile spikes were measured in the middle row (4 m^2^ areas) at the seedling (GS21), jointing (GS31), and maturity (GS92) stages (Fischer, 1985). The maximum seedling number and fertile spikes were calculated as the ratios of the maximum seedling number to the initial seedling number and the fertile spike number to the initial seedling number, respectively.

Grain yield was determined in a harvested area of 4 m^2^ for each plot. Fertile spikes were counted in a harvest area of 4 m^2^, and grain number per spike was counted for 30 spikes in each of the three trials. The grains from each plot was air-dried, weighed, and held for moisture determination using a DMC-700 digital moisture tester (Seedburo, Chicago, IL, USA). The 1,000-kernel weight was measured at a kernel moisture content of 13.5%. The grains from each plot were dried and ground into powder and passed through a 0.15-mm screen. The harvest index was calculated as the ratio of grain yield to above-ground dry matter yield at maturity.

#### NUE-related traits

Thirty wheat plants were collected consecutively from the middle row of each plot at anthesis (GS65) and physiological maturity to measure N content and NUE-related traits. The plant samples were separated into stems, green leaves, yellow leaves, spikes (glumes combined with rachilla, excluding grains), and grains. The segmented organ was oven-dried at 105°C for 30 min and then dried to a constant weight at 80°C for at least 72 h. The N content of the organs was determined using the Kjeldahl method (Kjeldahl, 1883). Soil N supply was the sum of potential N supply from soil and fertilizer. Potential N supply from the soil at sowing was estimated using the initial available N and bulk density. Total above-ground N accumulation was calculated as the product of above-ground dry matter and corresponding N content. NUE is defined as the average grain yield produced per kg of N supply and it can be subdivided into NUpE and NUtE. NUpE was calculated by dividing total above-ground N accumulation at harvest by soil N supply (Equation 1). NUtE is expressed as kilograms of grain yield per kilogram of total above-ground N accumulation (Equation 2). NHI was determined as the ratio of N accumulation in grains to total above-ground N accumulation at maturity (Equation 3). N partitioning was determined as the ratio of N accumulation at a specific organ (i.e., stems, green leaves, and spikes) to total above-ground N accumulation at anthesis. Post-anthesis N uptake was the difference between above-ground N accumulation at anthesis and maturity. N remobilization was calculated as the difference between above-ground N accumulation at anthesis and N accumulation in vegetative organs at maturity, including stems, leaves, and spikes. The remobilized dry matter (DM) and N were the DM and N lost from the vegetative organs, which was assumed to be remobilized to the developing organs.


(1) $$\begin{array}{l} \begin{array}{l} \text{NUpE }({\text{kg kg}}^{-1})=\text{Total above-ground N accumulation }/ \end{array}\\ \;\begin{array}{l} \left(\text{N fertilizer }+\text{ Soil mineral N}\right) \end{array} \end{array}$$

(2) $$\begin{array}{l} \begin{array}{l} \text{NUtE }({\text{kg kg}}^{-1})\;=\text{ Grain yield / Total above-ground} \end{array}\\ \begin{array}{l} \text{ N accumulation} \end{array} \end{array}$$

(3) $$\begin{array}{l} \begin{array}{l} \text{NHI }=\text{ N accumulation in grains }/\text{ Total above-ground} \end{array}\\ \begin{array}{l} \text{ N accumulation at maturity} \end{array} \end{array}$$

(4) $$\begin{array}{l} \text{N remobilization = Total above-ground N accumulation}\\ \begin{array}{l} \text{ at anthesis }-\text{Total above-ground N accumulation at}\\ \begin{array}{l} \text{ maturity excluding grains N} \end{array}\; \end{array} \end{array}$$

(5) $$\begin{array}{l} \text{N remobilization efficiency }=\text{ Remobilized N }/\\ \begin{array}{l} \text{ Total above-ground N at anthesis} \end{array} \end{array}$$

#### Grain protein quality-related traits

Grain protein content was measured as grain N content × 5.7. The wet gluten content and gluten index were measured according to gluten index method (Sekularac et al., 2018) using a Perten Glutomatic 2200 Instrument and a Perten 2015 Centrifuge (Perten Instruments AB, Hägersten, Sweden).

### Statistical analysis

Analysis of variance (ANOVA) was used to assess the effects of environment (E), genotype (G), year (Y), and their interactions (E × G, E × Y, G × Y, and E × G × Y). Statistical comparisons were considered significant at *P* < 0.05 or *P* < 0.01. Genotype plus genotype × environment (GGE) biplots were used to evaluate the productivity and sustainability of nine wheat genotypes in two wheat cropping systems. Principal component analysis (PCA) was used to evaluate relationships among NUE, yield, and protein quality-related traits. The contribution of key indicators to genetic variations in yield, NUpE, and NUtE were evaluated using dominance analysis. Associations among key indicators were evaluated using Pearson's correlation analysis.

## Results

### Genetic variations in grain yield, protein quality, and NUE-related traits

**Figure 2:**
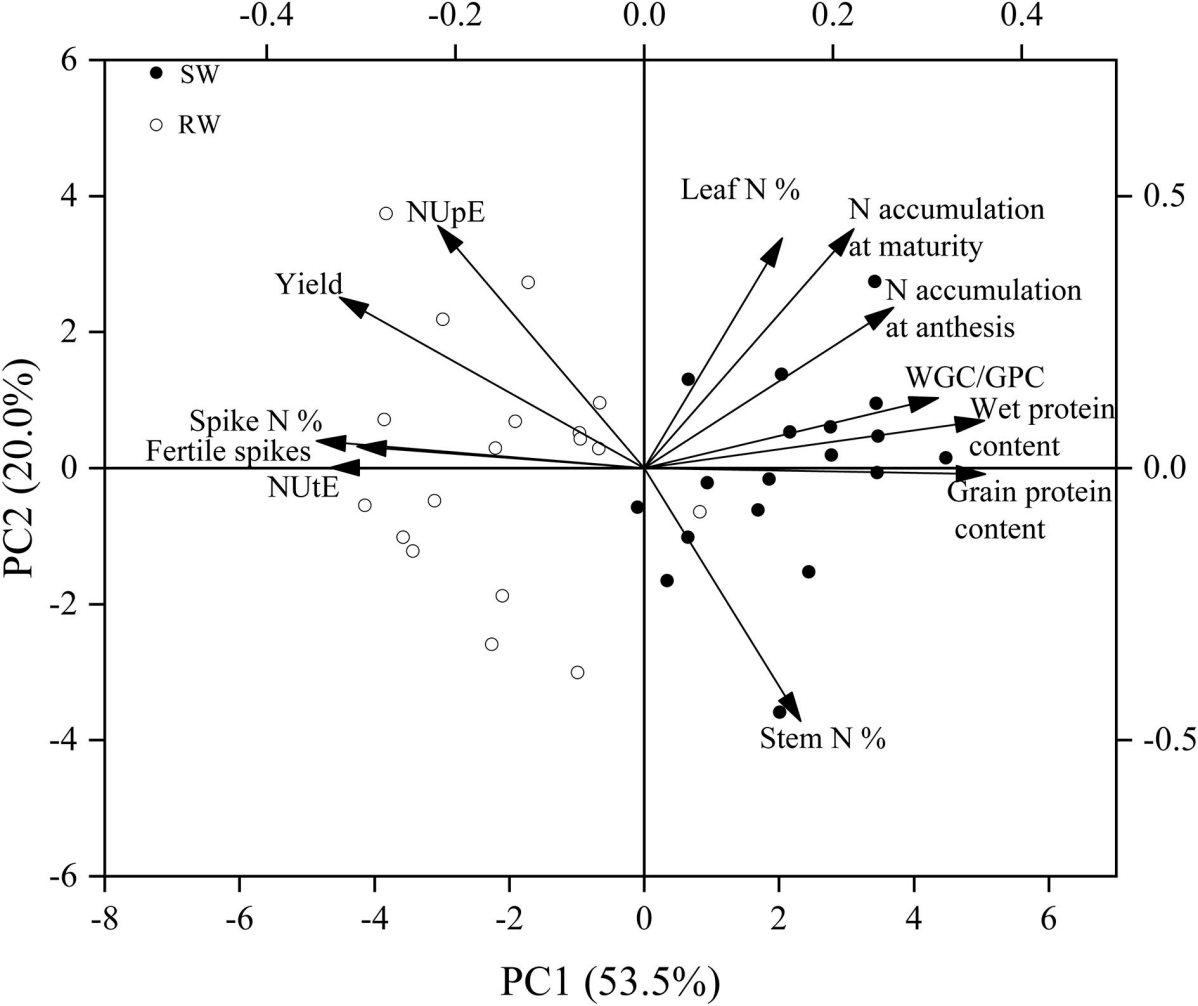
The principal component analysis (PCA) on yield, grain protein quality, and nitrogen efficiency and related traits in two wheat cropping systems and two cropping years. All values were measured with three replications (*n* = 3), and the two-year data were pooled. SW, soybean-wheat cropping system; RW, rice-wheat cropping system; WGC/GPC, Wet gluten content/grain protein content; N, nitrogen; NUpE, N uptake efficiency; NUtE, N utilization efficiency.

The wheat cropping systems affected most traits in nine wheat genotypes, except grain number per spike, 1,000-kernel weight, gluten index, and N remobilization efficiency. Post-anthesis N uptake was mainly affected by genotype. The 1,000-kernel weight, N remobilization efficiency, NHI, and NUtE in 2019–20 cropping year were markedly higher than those in 2018–19 cropping year. Meanwhile, significantly higher grain number per spike and gluten index in 2019–20 cropping year in comparison to 2018–19 cropping year were recorded only in the SW cropping system (Table 2). Higher grain yield, fertile spikes, NUpE, NUtE, and spike N% at anthesis were recorded in the RW cropping system, while higher grain protein content, wet gluten content, N accumulation at maturity, stem N% at anthesis, and leaf N% at anthesis were recorded in the SW cropping system (Figure 2).

**Table 2:** Combined ANOVA on variations in grain yield, protein quality, nitrogen efficiency, and related traits in two wheat cropping systems and two cropping years.

**Parameters**	**Mean values**				***F*** **-value**						
	**2018–19**		**2019–20**								
	**SW**	**RW**	**SW**	**RW**	**E**	**G**	**Y**	**E × G**	**E × Y**	**G × Y**	**E × G × Y**
Yield (t ha^−1^)	4.4c	6.6b	4.7c	7.6a	6522^**^	310^**^	480^**^	143^**^	116^**^	78^**^	138^**^
Fertile spikes (number per plant)	1.3c	1.9a	1.2c	1.7b	2547^**^	98^**^	258^**^	32^**^	31^**^	24^**^	29^**^
Grain number (number per spike)	39b	42ab	43a	46a	140^**^	183^**^	293^**^	17^**^	2^ns^	63^**^	17^**^
1,000-kernel weight (g)	49c	47d	52b	56a	26^**^	183^**^	1153^**^	23^**^	341^**^	6^**^	11^**^
Grain weight (g per spike)	1.9c	2.0c	2.2b	2.5a	187^**^	180^**^	1088^**^	16^**^	102^**^	46^**^	26^**^
Grain protein content (%)	12.0a	9.6c	11.4b	9.0d	3325^**^	470^**^	6^**^	25^**^	0^ns^	29^**^	17^**^
Wet gluten content (%)	24.2a	15.0b	24.5a	15.3b	4790^**^	470^**^	6^**^	25^**^	0^**^	36^**^	53^**^
Gluten index (%)	78b	89a	90a	91a	216^**^	310^**^	322^**^	53^**^	184^**^	116^**^	58^**^
Wet gluten content/ grain protein content	2.0a	1.5b	2.1a	1.7b	890^**^	159^**^	72^**^	30^**^	0^ns^	20^**^	26^**^
N accumulation at anthesis (10^−2^g per shoot)	4.5a	3.7b	4.9a	4.0b	823^**^	155^**^	168^**^	13^**^	0^ns^	40^**^	30^**^
Post anthesis N uptake (10^−2^g per shoot)	0.6a	0.7a	0.5a	0.8a	97^**^	182^**^	0^ns^	22^**^	8^**^	6^**^	45^**^
N accumulation at maturity (10^−2^g per shoot)	5.1ab	4.4c	5.4a	4.8bc	361^**^	119^**^	121^**^	6^**^	1^ns^	30^**^	18^**^
Stem N at anthesis (%)	39a	36b	39a	37b	109^**^	66^**^	3^ns^	11^**^	1^ns^	13^**^	5^**^
Leaf N at anthesis (%)	40a	36c	38ab	37bc	246^**^	94^**^	8^**^	8^**^	55^**^	27^**^	18^**^
Spike N at anthesis (%)	20b	27a	21b	26a	1246^**^	38^**^	2^ns^	20^**^	62^**^	13^**^	26^**^
N remobilization efficiency (%)	75b	71c	79a	80a	129^**^	41^**^	2232^**^	37^**^	357^**^	53^**^	60^**^
NHI (%)	78b	76c	81a	83a	0^ns^	129^**^	3396^**^	82^**^	534^**^	108^**^	24^**^
NUpE (kg kg^−1^)	0.47b	0.58a	0.47b	0.57a	1153^**^	118^**^	1^ns^	70^**^	0^ns^	65^**^	81^**^
NUtE (kg kg^−1^)	38d	46b	41c	53a	3384^**^	201^**^	1034^**^	26^**^	164^**^	48^**^	10^**^

All data were the mean values of nine wheat genotypes in each cropping year (n = 9).

E, environment; G, genotype; Y, year; E × G, interaction between environment and genotype; E × Y, interaction between environment and year; G × Y, interaction between genotype and year; E × G × Y, interaction between environment, genotype, and year; SW, soybean-wheat cropping system; RW, rice-wheat cropping system; N, nitrogen; NUpE, N uptake efficiency; NUtE, N utilization efficiency; NHI, N harvest index.

^ns^represents non-significant at *P* > 0.05, while * and

** represent significant at *P* < 0.05 and *P* < 0.01 from ANOVA, respectively.

Different lowercase letters indicate significant difference at P < 0.05.

### Grain yield and yield-related traits

Dominance analysis revealed that in 2018–19 cropping year, fertile spikes, grain number per spike, 1,000-kernel weight in SW and RW cropping systems contributed by 12.6, 48.0, and 37.0% and 25.6, 48.6, and 24.4% of grain yield variation, respectively. Meanwhile, in 2019–20 cropping year, fertile spikes, grain number per spike, 1,000-kernel weight in SW and RW cropping systems contributed by17.1, 70.2, and 9.4% and 43.7, 20.4, and 35.3% of grain yield variation, respectively. Averaged across the two cropping years, grain number per spike showed a more significant and greater contribution to grain yield variation than other yield components in the SW cropping system, while fertile spikes and 1,000-kernel weight showed more outstanding contribution to grain yield variation in RW cropping system.

**Figure 3:**
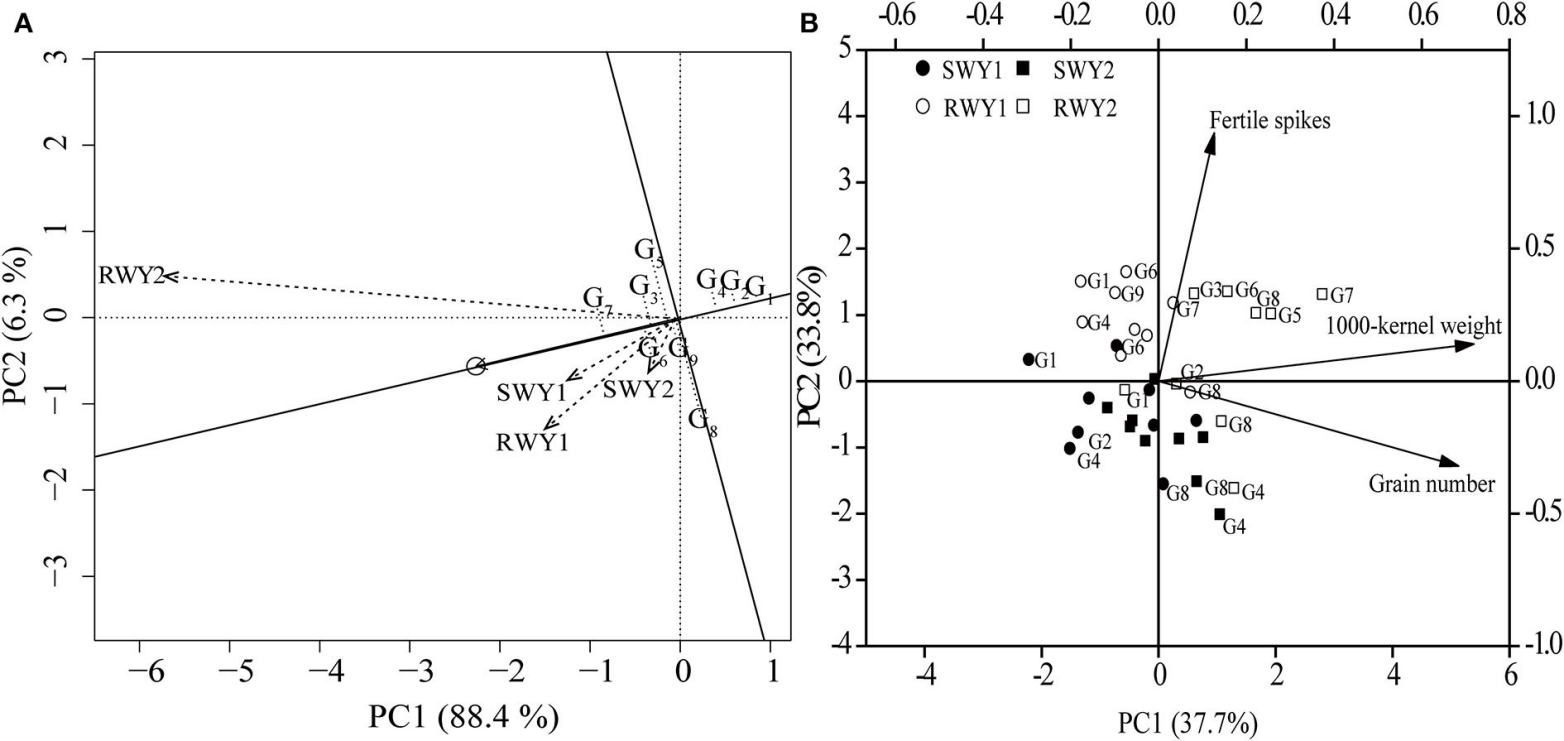
The genotype main effects and genotype × environment interaction analysis **(A)** on mean yield, yield stability, and principal component analysis (PCA) **(B)** on yield components of nine wheat genotypes in two wheat cropping systems and two cropping years. All values were measured with three replications (*n* = 3). The vectors represent the loading scores of variables for PC1 and PC2. SW, soybean-wheat cropping system; RW, rice-wheat cropping system; G, genotype; Y1, 2018–19 cropping year; Y2, 2019–20 cropping year.

When averaged across the two cropping systems and two cropping years, SM-482, CM-39, and NM-101 showed stably low grain yield, while CY-25 showed the highest grain yield, which was primarily attributed to its highest 1,000-kernel weight. MM-51 and MY-26 showed low-yield sustainability due to their low fertile spikes in the RW cropping system in 2019–20 cropping year and low grain number per spike in the SW cropping system in 2018–19 cropping year, respectively (Table 3 and Figure 3).

**Table 3:** The grain yield and yield components of nine wheat genotypes in two wheat cropping systems and two cropping years.

**Genotype**	**Yield**				**Fertile spikes**				**Grain number**				**1,000-kernel weight**			
	**(t ha** ^**−1**^ **)**				**(number per plant)**				**(number per spike)**				**(g)**			
	**2018–19**		**2019–20**		**2018–19**		**2019–20**		**2018–19**		**2019–20**		**2018–19**		**2019–20**	
	**SW**	**RW**	**SW**	**RW**	**SW**	**RW**	**SW**	**RW**	**SW**	**RW**	**SW**	**RW**	**SW**	**RW**	**SW**	**RW**
SM-482	3.3e	5.9d	4.3d	4.3g	1.4a	2.0b	1.3ab	1.3d	28e	37e	38f	35f	47d	45d	49ef	53de
CM-39	3.9d	6.0d	4.1d	5.5f	1.2b	1.7d	1.1cd	1.4c	38c	43b	38f	40e	46d	46bc	52cd	55c
CM-66	4.6bc	6.7b	4.8c	9.2b	1.4a	2.0b	1.3ab	1.9b	39bc	41bc	43cd	47c	46d	46bc	48f	57b
NM-101	3.8d	5.6e	5.5a	6.4e	1.2bc	1.8c	1.1d	1.2d	41b	38de	58a	57a	43e	45cd	48f	50f
MY-26	4.6bc	6.3c	4.1d	9.1bc	1.2bc	1.7d	1.1d	1.8b	40b	40cd	39ef	45d	53b	51a	53bc	61a
CM-81	4.5c	7.4a	5.1b	8.9c	1.4a	2.1a	1.4a	2.0a	33d	40cd	38f	46cd	53b	47b	54b	55cd
CY-25	4.9a	7.5a	4.7c	11.3a	1.2bc	1.9c	1.1d	2.0a	41b	43b	41de	51b	56a	51a	59a	62a
MM-51	4.8ab	7.4a	4.9bc	6.4e	1.1c	1.7d	1.1d	1.4c	49a	54a	50b	49b	48c	46bc	51de	53e
CM-104	5.1a	6.5bc	5.1b	7.5d	1.4a	1.8c	1.2bc	1.8b	40b	42bc	44c	41e	52b	47b	53bc	55cd
Mean	4.7	6.6	4.7	7.6	1.3	1.9	1.1	1.6	39	42	43	49	49	47	52	56
*F*-value	42^**^	59^**^	33^**^	423^**^	17^**^	32^**^	13 ^**^	163^**^	74^**^	46^**^	60^**^	125^**^	166^**^	22^**^	34^**^	67^**^

All values were measured with three replications (n = 3).

SW, soybean-wheat cropping system; RW, rice-wheat cropping system.

* and

** represent significant at *P* < 0.05 and *P* < 0.01 from ANOVA, respectively.

Different lowercase letters indicate significant difference at P < 0.05.

### Grain protein quality-related traits

Dominance analysis revealed that grain protein content, wet gluten content/grain protein content, gluten index in SW and RW wheat cropping systems contributed by 40.1, 52.2, and 7.2% and 27.6, 58.2, and 13.6% of variation in wet gluten content. Wet gluten content/grain protein content accounted for a more significant variation in wet gluten content. However, a significantly negative correlation between wet gluten content/grain protein content and gluten index (*R*^2^ = 0.39, *P* < 0.01) was observed in the RW cropping system. Regarding overall performance over the two cropping systems and two cropping years, CM-39, MY-26, and CY-25 showed a higher wet gluten content, whereas the gluten index of CM-39 alone exceeded 90% (Table 4).

**Table 4:** The grain protein quality-related traits of nine wheat genotypes in two wheat cropping systems and two cropping years.

**Genotype**	**Grain protein content**				**Wet gluten content**				**Wet gluten content/**				**Gluten index**			
	**(%)**				**(%)**				**grain protein content**				**(%)**			
	**2018–19**		**2019–20**		**2018–19**		**2019–20**		**2018–19**		**2019–20**		**2018–19**		**2019–20**	
	**SW**	**RW**	**SW**	**RW**	**SW**	**RW**	**SW**	**RW**	**SW**	**RW**	**SW**	**RW**	**SW**	**RW**	**SW**	**RW**
SM-482	12.8b	10.0bc	12.3a	9.2bc	25.7d	13.7e	24.5c	18.2b	2.0cd	1.4e	2.0cde	2.0b	83c	93bc	93bc	83c
CM-39	14.8a	10.6a	12.3a	10.5a	31.5a	18.8c	28.8b	23.5a	2.1bc	1.8c	2.3b	2.3a	95ab	99a	99a	97a
CM-66	10.8d	8.4f	11.7b	8.5de	17.7f	8.6g	23.2c	12.1e	1.7e	1.0g	2.0def	1.5e	79d	97ab	92bc	96a
NM-101	12.9b	10.6a	10.9c	9.0c	29.3b	17.0d	23.4c	17.6bc	2.3ab	1.6d	2.1c	1.9b	47g	94bc	94bc	89b
MY-26	12.0c	10.1b	11.5b	9.5b	28.6b	23.3a	28.9b	17.1c	2.4a	2.3a	2.5a	1.8c	58e	55e	85e	88b
CM-81	10.9d	8.6ef	10.6cd	8.2f	17.1f	14.0e	19.6e	12.6e	1.6e	1.6d	1.8f	1.6de	94b	92c	88d	88b
CY-25	11.9c	9.8c	12.3a	9.3bc	27.0c	20.2b	30.8a	15.2d	2.3ab	2.1b	2.5a	1.6d	50f	75d	72f	84c
MM-51	10.7d	8.7e	10.5d	8.2ef	20.1e	6.9h	21.4d	10.3g	1.9d	0.8h	2.0cd	1.2f	98a	98a	91cd	95a
CM-104	10.8d	9.5d	10.5cd	8.6d	20.8e	12.0f	19.6e	11.2f	1.9d	1.3f	1.9ef	1.3f	96ab	97ab	95b	95a
Mean	12	9.6	11.4	9.0	24.2	14.9	24.5	15.3	2.0	1.5	2.1	1.7	78	89	90	91
*F*-value	91^**^	58^**^	31^**^	62^**^	192^**^	167^**^	72 ^**^	251^**^	30^**^	135^**^	26^**^	71^**^	517^**^	101^**^	50^**^	21^**^

All values were measured with three replications (n = 3).

SW, soybean-wheat cropping system; RW, rice-wheat cropping system.

* and

** represent significant at *P* < 0.05 and *P* < 0.01 from ANOVA, respectively.

Different lowercase letters indicate significant difference at P < 0.05.

### Critically NUE-related traits associated with simultaneous selection for high grain yield and good protein quality

**Figure 4:**
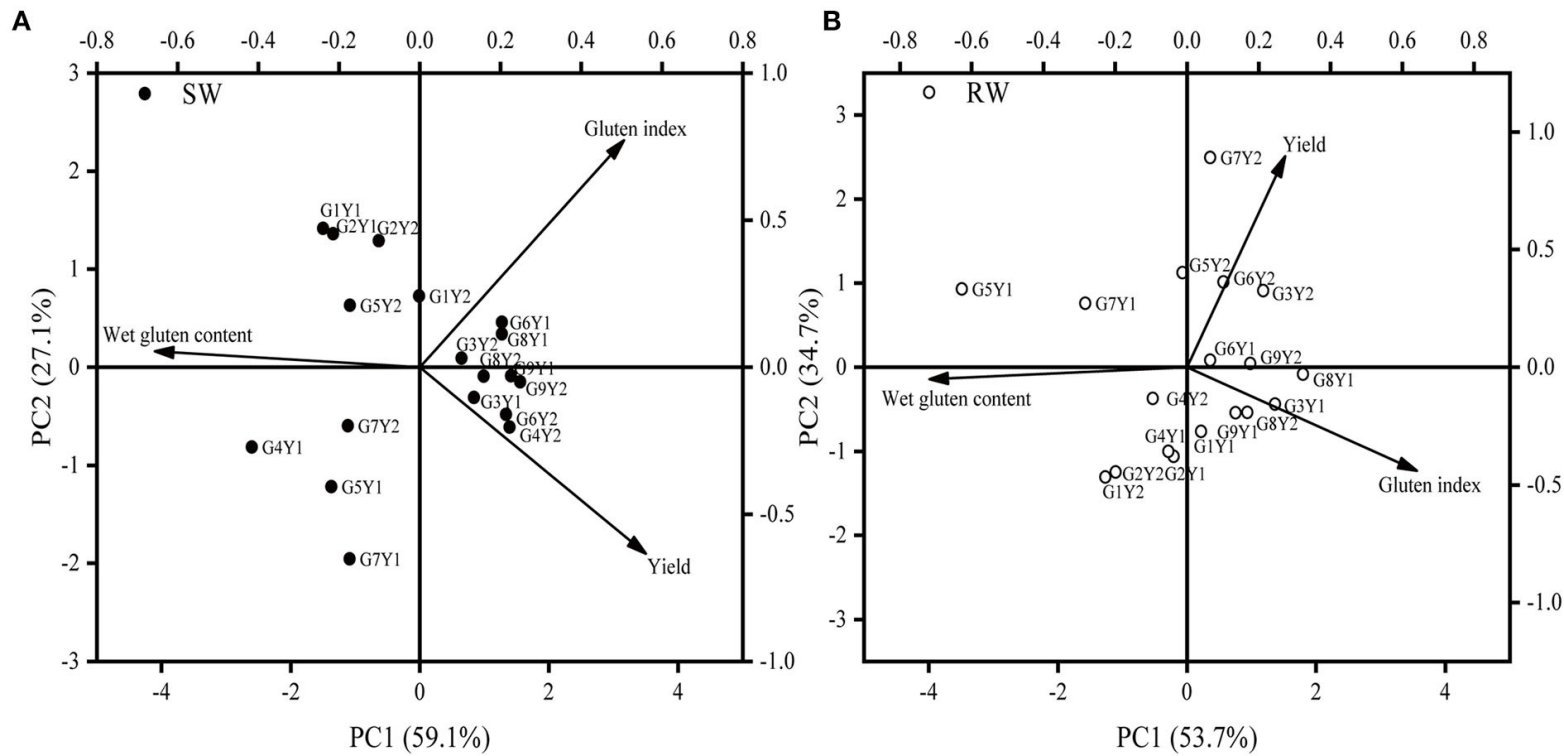
The PCA on yield and grain protein quality of nine wheat genotypes in two wheat cropping systems and two cropping years. All values were measured with three replications (*n* = 3). The vectors represent the loading scores of variables for PC1 and PC2. SW, soybean-wheat cropping system; RW, rice-wheat cropping system; G, genotype; Y1, 2018−19 cropping year; Y2, 2019–20 cropping year.

Results from PCA revealed that MY-26 and CY-25 achieved high grain yield and wet gluten content in the two wheat cropping systems in 2018–19 cropping year, which was associated with high 1,000-kernel weight, post- anthesis N uptake, N accumulation at maturity, and low gluten index (Table 5 and Figure 4). It was noteworthy that CY-25 with low plant height showed high NHI (Table 6). In addition, the wet gluten content of NM-101 in 2019–20 cropping year was higher than that of MM-51 at an equivalently high level of grain number per spike, which was mainly related to its higher plant height, flag leaf area (data not shown), and N accumulation at anthesis.

**Table 5:** The N uptake-related traits of nine wheat genotypes in two wheat cropping systems and two cropping years.

**Cropping** **years**	**Genotype**	**Plant** **height** **(cm)**		**Days from sowing** **to flowering** **(d)**		**N accumulation** **at anthesis** **(10**^**−2**^**g per shoot)**		**Post anthesis** **N uptake** **(10**^**−2**^**g per shoot)**		**N accumulation** **at maturity** **(10**^**−2**^**g per shoot)**		**NUpE** **(kg kg**^**−1**^**)**	
		**SW**	**RW**	**SW**	**RW**	**SW**	**RW**	**SW**	**RW**	**SW**	**RW**	**SW**	**RW**
2018–19	SM-482	80	90	143	145	3.2f	3.1d	0.6c	0.7d	3.8e	3.8d	0.39e	0.54c
	CM-39	90	94	147	148	5.7a	4.1b	0.2de	0.7d	5.9ab	4.8ab	0.53a	0.58b
	CM-66	89	89	147	148	4.0d	3.2d	0.3de	0.4e	4.3d	3.7d	0.44cd	0.51c
	NM-101	96	103	147	148	5.1b	4.0b	0.1e	0.4e	5.2c	4.5c	0.45cd	0.58b
	MY-26	90	92	140	144	5.2b	3.7c	0.8b	1.2b	6.0a	4.8a	0.52a	0.60ab
	CM-81	84	84	140	143	3.6e	2.9d	0.4d	0.9c	4.0e	3.8d	0.42d	0.59b
	CY-25	86	90	143	145	4.4c	3.2d	1.5a	1.4a	5.9ab	4.6bc	0.50a	0.62a
	MM-51	89	93	143	145	5.0b	4.5a	0.6bc	0.3ef	5.7b	4.9a	0.47bc	0.58b
	CM-104	89	95	140	144	4.5c	4.3ab	0.6c	0.2f	5.0c	4.6bc	0.50ab	0.60ab
	Mean	88	92	143	146	4.5	3.7	0.6	0.7	5.1	4.4	0.47	0.58
	*F*-value	\	\	\	\	108^**^	32^**^	52^**^	54^**^	63^**^	33 ^**^	20^**^	14^**^
2019–20	SM-482	88	77	131	135	4.6e	2.8g	0.3de	0.7c	5.0ef	3.6f	0.46c	0.33h
	CM-39	86	80	135	142	5.3bc	4.7b	0.1f	0.3e	5.4cde	5.0c	0.45c	0.49f
	CM-66	94	80	135	139	5.0cd	4.5c	0.4de	0.4de	5.5cd	4.9c	0.52a	0.67c
	NM-101	101	90	135	139	6.1a	5.1a	0.5cd	0.2e	6.5a	5.4b	0.52a	0.48f
	MY-26	87	84	128	135	4.5e	4.1d	0.8ab	1.3b	5.2de	5.4b	0.41d	0.72b
	CM-81	84	75	128	132	3.6f	3.9e	0.9a	0.5d	4.5f	4.4de	0.46c	0.61d
	CY-25	87	84	131	139	5.4b	3.9e	0.7ab	2.2a	6.1ab	6.1a	0.49b	0.87a
	MM-51	92	91	135	142	5.1bc	3.7f	0.6bc	0.7c	5.7bc	4.5d	0.44c	0.44g
	CM-104	91	88	128	138	4.7de	3.7f	0.3e	0.5d	5.0e	4.2e	0.44c	0.55e
	Mean	90	83	132	138	4.9	4.0	0.5	0.8	5.4	4.8	0.47	0.57
	*F*-value	\	\	\	\	41^**^	120^**^	18^**^	172^**^	18^**^	119 ^**^	22^**^	209^**^

All values were measured with three replications (n = 3).

SW, soybean-wheat cropping system; RW, rice-wheat cropping system; N, nitrogen; NUpE, N uptake efficiency.

* and

** represent significant at *P* < 0.05 and *P* < 0.01 from ANOVA, respectively.

Different lowercase letters indicate significant difference at P < 0.05.

**Table 6:** The N utilization-related traits of nine wheat genotypes in two wheat cropping systems and two cropping years.

**Genotype**	**N remobilization efficiency**				**NHI**				**NUtE**			
	**(%)**				**(%)**				**(kg kg** ^**−1**^ **)**			
	**2018–19**		**2019–20**		**2018–19**		**2019–20**		**2018–19**		**2019–20**	
	**SW**	**RW**	**SW**	**RW**	**SW**	**RW**	**SW**	**RW**	**SW**	**RW**	**SW**	**RW**
SM-482	73f	72c	79de	80bc	78c	77c	80e	84b	34de	44d	37d	52cde
CM-39	76cd	72c	78e	80bc	76d	76c	79f	82d	29f	41f	37d	45f
CM-66	77b	73c	74f	80bc	79b	77c	77g	82d	42b	52a	37d	55b
NM-101	76c	69e	81bc	85a	77d	72e	82d	85a	34e	39g	43b	54bcd
MY-26	71g	66f	78e	80c	74e	75d	81e	85ab	35d	42ef	40c	51e
CM-81	81a	67f	78de	81bc	82a	75d	83c	83c	43a	50b	45a	58a
CY-25	76cd	75b	82b	76d	82a	82a	84b	85ab	39c	48c	39c	52de
MM-51	75de	77a	80cd	81b	78c	78b	82cd	85ab	41b	51ab	45a	59a
CM-104	75e	70d	84a	80bc	78c	71e	85a	82cd	41b	43de	46a	54bc
Mean	75	71	79	80	78	76	81	84	38	46	41	53
*F*-value	97^**^	70^**^	41^**^	20^**^	80^**^	222^**^	85^**^	21^**^	164^**^	106^**^	46^**^	41^**^

All values were measured with three replications (n = 3).

SW, soybean-wheat cropping system; RW, rice-wheat cropping system; N, nitrogen; NHI, N harvest index; NUtE, N utilization efficiency.

* and

** represent significant at *P* < 0.05 and *P* < 0.01 from ANOVA, respectively.

Different lowercase letters indicate significant difference at P < 0.05.

### Associations among grain yield, protein quality, and NUE and related traits in the two wheat cropping systems

The grain yield in the RW cropping system was higher than that in the SW cropping system, due to higher fertile spikes (Table 3) and spike N partitioning at anthesis (Table 7), while lower grain protein content (Table 4) in the RW cropping system was mainly related to lower N accumulation at maturity (Table 5), rather than NHI (Table 6). The low gluten index of MY-26 and CY-25 (Table 4) in two wheat cropping systems were mainly associated with high 1,000-kernel weight and post-anthesis N uptake, while the low gluten index of NM-101 and MY-26 in the SW cropping system in 2018–19 cropping year (Table 4) were mainly related to low NHI caused by low grain number per spike and N remobilization efficiency.

**Table 7:** The N partitioning at anthesis of nine wheat genotypes in two wheat cropping systems and two cropping years.

**Genotype**	**N partitioning at anthesis (%)**											
	**2018–19**						**2019–20**					
	**SW**			**RW**			**SW**			**RW**		
	**Stem**	**Leaf**	**Spike**	**Stem**	**Leaf**	**Spike**	**Stem**	**Leaf**	**Spike**	**Stem**	**Leaf**	**Spike**
SM-482	46a	33g	20bc	43a	30e	25cde	45a	35d	19e	39b	33cd	27b
CM-39	43b	37f	19cde	36bc	37c	26cde	41b	38bc	19e	41ab	33cd	24c
CM-66	38d	41cd	19bcd	36cd	38abc	24e	38cd	39b	19e	33cd	42a	23c
NM-101	37e	42bc	20b	33d	39ab	25de	38c	37cd	23b	35c	34cd	30a
MY-26	32f	49a	18de	33d	38bc	27c	35e	43a	22c	34cd	44a	21d
CM-81	40c	40de	19cde	34cd	32e	32a	36de	38b	24a	34cd	37b	27b
CY-25	41c	39e	18e	35cd	34d	29b	39c	39b	21cd	32d	38b	29a
MM-51	41c	38ef	20bc	39b	33d	26cd	42b	35d	21d	42a	35c	22d
CM-104	31f	44b	24a	34cd	39a	25cde	38cd	39b	22c	39b	32d	27b
Mean	39	40	20	36	36	27	39	38	21	37	37	26
*F*-value	121^**^	54^**^	17^**^	12^**^	46^**^	16^**^	21^**^	14^**^	33^**^	16^**^	38^**^	36^**^

All values were measured with three replications (n = 3).

SW, soybean-wheat cropping system; RW, rice-wheat cropping system; N, nitrogen.

* and

** represent significant at *P* < 0.05 and *P* < 0.01 from ANOVA, respectively.

Different lowercase letters indicate significant difference at P < 0.05.

**Figure 5:**
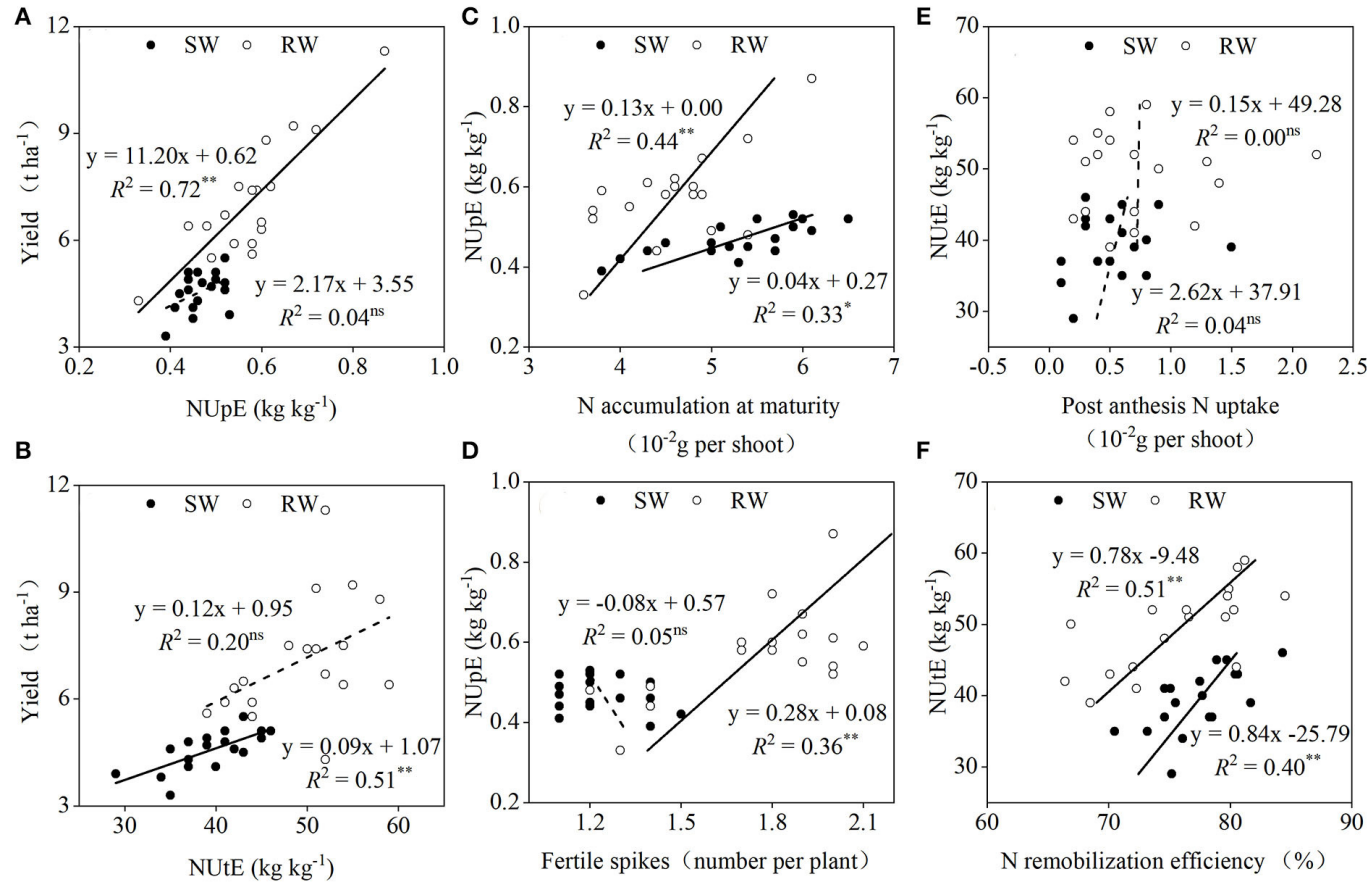
Relationships between plant height and yield **(A)**, harvest index **(B)**; between yield and harvest index **(C)**; between N accumulation at anthesis and plant height **(D)**, days from sowing to flowering **(E)**, N accumulation rate **(F)** in two wheat cropping systems. All values were measured with three replications (*n* = 3), two-year data were pooled. SW, soybean-wheat cropping system; RW, rice-wheat cropping system; N, nitrogen. ^ns^represents non-significant at *P* > 0.05, while ^*^ and ^**^represent significant at *P* < 0.05 and *P* < 0.01 from ANOVA, respectively. Solid and dashed lines indicate significant and insignificant differences at *P* < 0.05 and *P* > 0.05, respectively.

No significant correlation between grain yield and plant height was observed in both wheat cropping systems (Figure 5A). When averaged across the two cropping years, the genetic gain of grain yield was mainly related to the improvement of harvest index, especially in the SW cropping system (Figure 5C). However, the negative correlation between plant height and harvest index was only recorded in the RW cropping system (Figure 5B). N accumulation at anthesis was mainly associated with N accumulation rate (Figure 5F), rather than days from sowing to flowering (Figure 5E) in both wheat cropping systems. In addition, N accumulation at anthesis showed a significantly positive correlation with plant height in the SW cropping system (Figure 5D).

**Figure 6:**
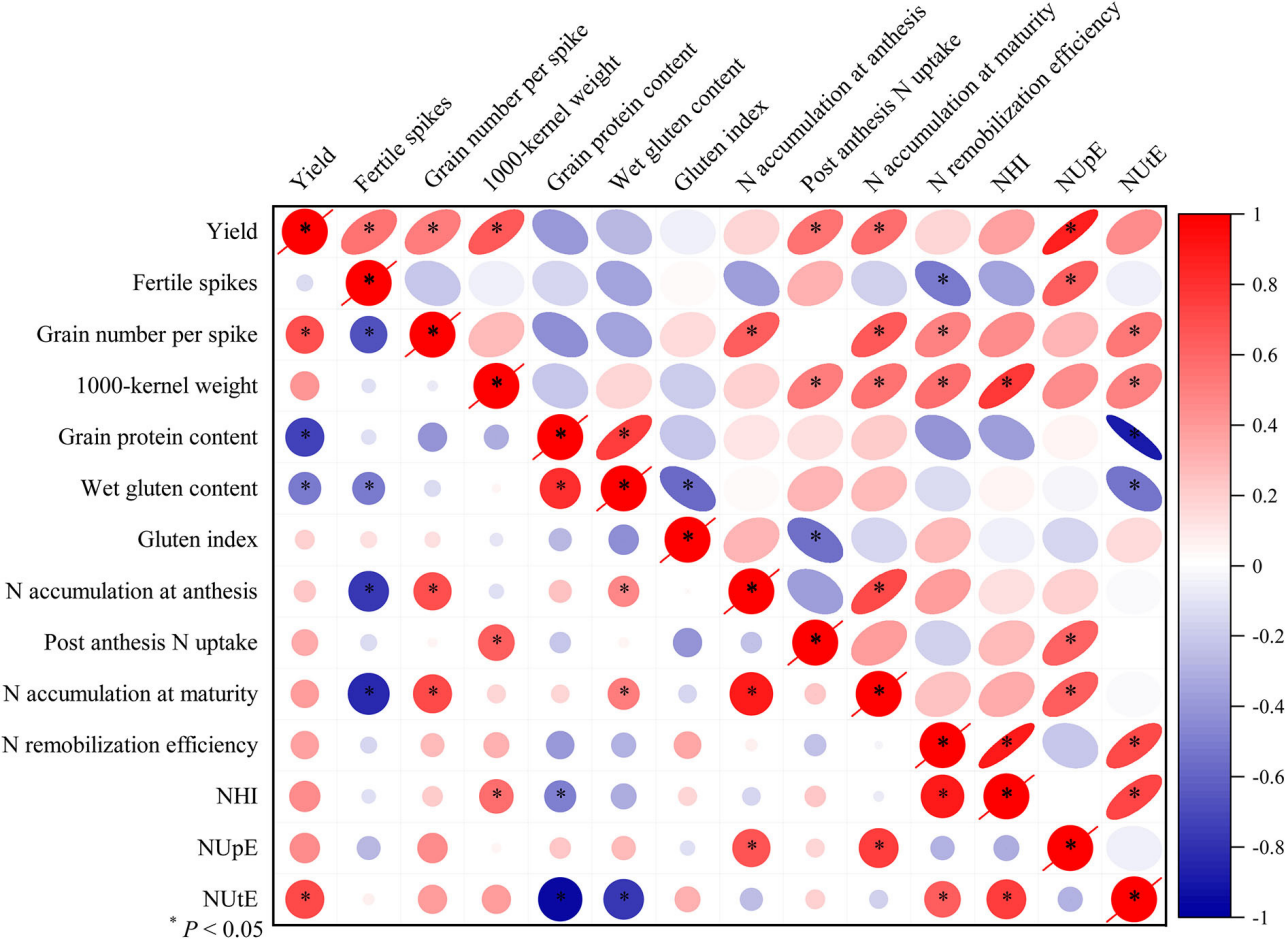
The Pearson's correlation analysis on yield, grain protein quality, and NUE-related traits. All values were measured with three replications (*n* = 3), two-year data were pooled. N, nitrogen; NUpE, N uptake efficiency; NUtE, N utilization efficiency; NHI, N harvest index; *R*, Pearson's *R*. *P < 0.05. Circles and ellipses represent SW and RW cropping systems, respectively.

Results from correlation analysis (Figure 6) revealed that when averaged across the two cropping years, grain yield in the SW cropping system were positively correlated with grain number per spike (*R*^2^ = 0.48, *P* < 0.01), NUtE (*R*^2^ = 0.51, *P* < 0.01) and negatively correlated with grain protein content (*R*^2^ = 0.52, *P* < 0.01). Grain yield in the RW cropping system were positively correlated with fertile spikes (*R*^2^ = 0.30, *P* < 0.05), grain number per spike (*R*^2^ = 0.26, *P* < 0.05), 1,000-kernel weight (*R*^2^ = 0.42, *P* < 0.01), post anthesis N uptake (*R*^2^ = 0.29, *P* < 0.05), N accumulation at maturity (*R*^2^ = 0.31, *P* < 0.05) and NUpE (*R*^2^ = 0.75, *P* < 0.01). In addition, wet gluten content was negatively correlated with fertile spikes in the RW cropping system (*R*^2^ = 0.26, *P* < 0.05) and gluten index in the RW cropping system (*R*^2^ = 0.32, *P* < 0.05).

**Figure 7:**
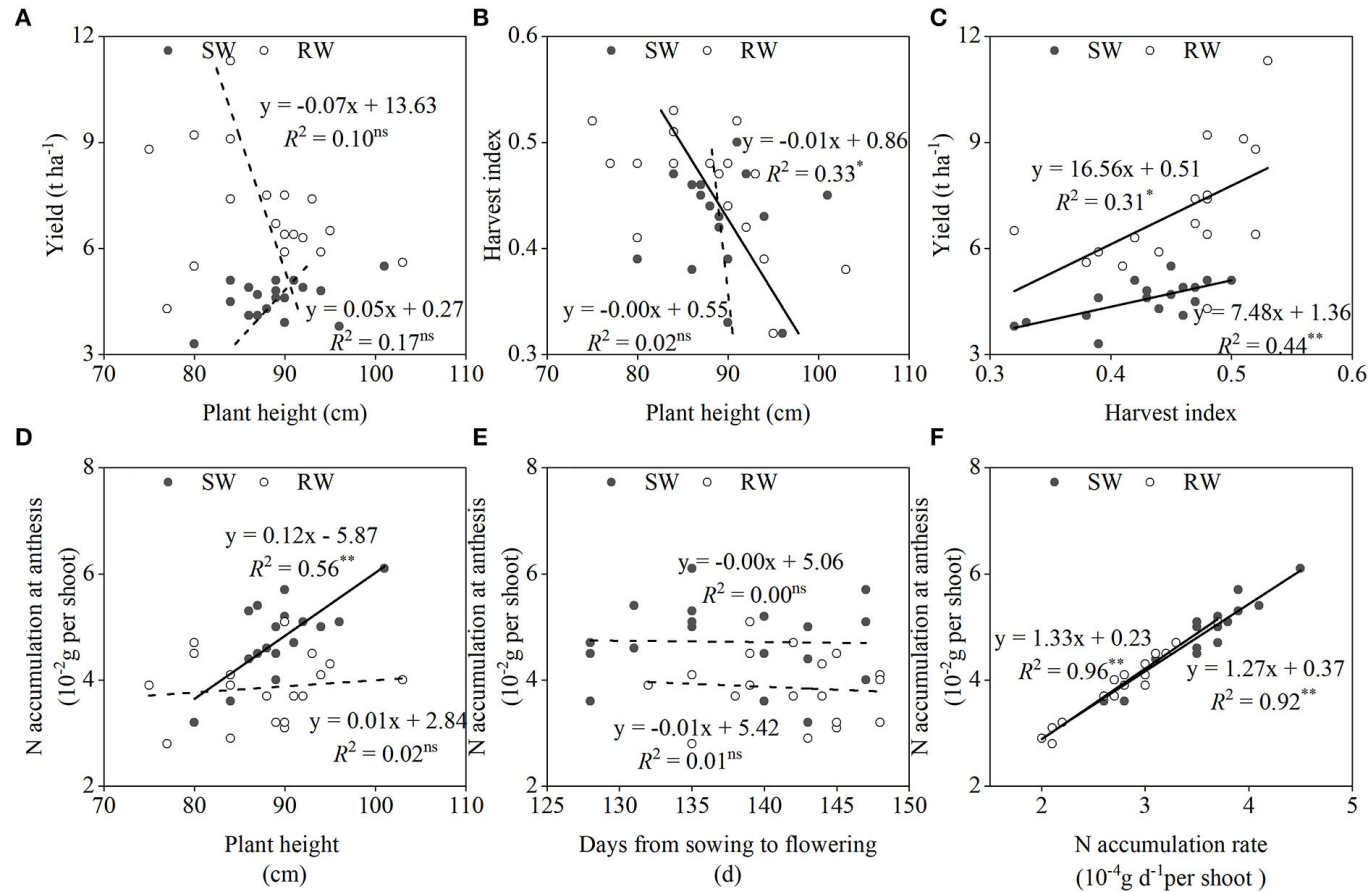
Relationships between yield and NUpE **(A)**, NUtE **(B)**; between NUpE and N accumulation at maturity **(C)**, fertile spikes **(D)**; between NUtE and post-anthesis N uptake **(E)**, N remobilization efficiency **(F)** in two wheat cropping systems. All values were measured with three replications (*n* = 3), two-year data were pooled. SW, soybean-wheat cropping system; RW, rice-wheat cropping system; N, nitrogen; NUpE, N uptake efficiency; NUtE, N utilization efficiency. ^ns^represents non-significant at *P* > 0.05, while ^*^ and ^**^represent significant at *P* < 0.05 and *P* < 0.01 from ANOVA, respectively. Solid and dashed lines indicate significant and insignificant differences at *P* < 0.05 and *P* > 0.05, respectively.

Dominance analysis revealed that when averaged across the two cropping years, NUpE (Figure 7A) and NUtE (Figure 7B) in the SW and RW cropping systems accounted for 34.3 and 65.2% and 77.6 and 22.1% of grain yield variations, respectively. The NUpE in the two wheat cropping systems showed a significant linear correlation with N accumulation at maturity (Figure 7C), whereas a significant linear correlation between NUpE and fertile spikes was only recorded in the RW cropping system (Figure 7D). In both wheat cropping systems, the NUtE showed an insignificant linear correlation with post-anthesis N uptake (Figure 7E) but a significant linear correlation with N remobilization efficiency (Figure 7F).

## Discussion

### NUpE and NUtE played different roles in grain yield variation in two wheat cropping systems due to varied water and N environment

In rotation systems, preceding crops significantly affect subsequent crop growth and development (Ibrahim et al., 2018; Ren et al., 2019). In the present study, significantly higher grain yield was observed in the RW cropping system rather than the SW cropping system, which can be attributed to variations in soil water and N conditions. Soil water deficiency is the primary limiting factor in the SW cropping system, and a decrease in soil water leads to reductions in N uptake, tiller population, fertile spikes, and ultimately decreases grain yield (Lin et al., 2020). However, in the present study, fertile spikes and grain number per spike explained greater variation of yield in RW and SW cropping systems, respectively, which was consistent with the finding that the contribution of yield components to yield varied in different ecological regions (Wu et al., 2014; Qin et al., 2015). These results also confirmed that under favorable soil conditions, the genetic gain of wheat yield was primarily attributed to fertile spikes, followed by grain number per spike and 1,000-kernel weight (Sobczyński et al., 2020). We found that grain number per spike is the limiting factor in the SW cropping system restricting grain yield in super high yield levels rather than fertile spikes, which was inconsistent with the study by Moragues et al. (2006) which observed that wheat grain yield was mainly determined by fertile spikes under water stress, and is in agreement with the finding that wheat grain yield was mainly contributed by main stem under rain fed or drought conditions (Elhani et al., 2007). In addition, our results also showed that the different contribution of fertile spikes to yield in RW and SW may be related to tiller occurrence (Samarah, 2005) and tiller survival (Sadras and Slafer, 2012), respectively.

The contribution of NUpE and NUtE to grain yield varied in the two wheat cropping systems, which was related to the variant contribution of yield components to grain yield. The NUpE represents the capability of roots to capture soil water and N from the soil (Hawkesford, 2014; Aziz et al., 2017; Wang et al., 2018). In this study, a higher total N uptake in the RW cropping system reduced the competitive advantage of the main stem, thus achieving higher fertile spikes (Guo et al., 2013). However, the NUpE explained more significant yield variation in the RW cropping system resulting from variation in fertile spikes, which was due to the reduction in maximum tiller population rather than tiller survival rate (Jalota et al., 2013). Future studies still require investigating the contribution of root morphological traits to the genetic variation of NUpE in RW cropping systems.

In contrast, soil water deficiency in the SW cropping system decreased the capability of roots to capture soil water and N (Harrison et al., 2011), resulting in the death of tillers when N demands increase after stem elongation stage (GS31). Therefore, NUtE in the SW cropping system explained more significant yield variation resulting from grain number per spike and (or) kernel weight (Driever et al., 2014; Jagadish et al., 2015; Prins et al., 2016). The results of this study also supported the above finding. The NUtE represents the capability of crops to convert the absorbed N into grain yield (De Oliveira Silva et al., 2020). Previous results showed that an increase in the grain number per spike was attributed to increased spike DM, fruiting efficiency, and reduced tiller population (Reynolds et al., 2012; Ittersum and Martin, 2016; Carolina et al., 2019). The introduction of tiller suppressor gene (tin) increased grain number per spike (Kumar et al., 2015), due to increased roots in deeper soil layers (Houshmandfar et al., 2020) and increased biomass partitioning to spike, which increased the fruiting efficiency, ultimately increasing the grain yield. This explanation is similar to previous results (Acreche et al., 2008; Lo Valvo et al., 2018). In our study, wheat genotypes with high grain number per spike also present high plant height, leaf area index (NM-101), and erect canopy structures (MM-51). These traits were reported as determining factors for improving the efficiency in the light interception and solar energy conversion and fruiting efficiency (Shearman et al., 2005; Reynolds et al., 2015; Chen et al., 2018). This is because wheat cultivars with high light energy interception and photosynthesis (Ghiglione et al., 2010) contributed to a higher differentiated and fertile florets (González et al., 2005; Ferrante et al., 2013). In addition, soil water deficiency in the SW cropping system decreased *P*_n_ resulting from non-stomatal limitation (Friso et al., 2004; Gómez-Bellot et al., 2020), primarily due to decreased rubisco activity (Bertheloot et al., 2008). The decrease in grain number per spike (Borrás et al., 2004) reduced the photosynthetic capacity of leaves (Reynolds et al., 2015) and kernel weight, indicating that grain yield in the SW cropping system was affected by both sink and source restrictions (Serrago and Alzueta, 2013). Another hypothesis is that sink improvement caused by the combined effect of grain number per spike and 1,000-kernel weight contributes to the biomass remobilization efficiency (Zhang et al., 2019), thereby improving the N remobilization efficiency. Therefore, further improvements in NUtE can be achieved by increasing post-anthesis N uptake and N remobilization efficiency.

### N accumulation at maturity and N remobilization affected grain protein quality

Grain protein content is one of the most important traits affecting wheat processing quality (Ma et al., 2019), and is the product of N accumulation at maturity, grain weight per spike, and NHI (Gaju et al., 2011; Marti and Slafer, 2014; Wang et al., 2017). In the present study, grain protein content in the SW cropping system was higher than that in the RW cropping system, due to higher N accumulation at maturity resulting from competitive advantage of the main stem to N resources (Tamang et al., 2017), which was in agreement with previous study (Zhang et al., 2017). In contrast, low grain protein content in the RW cropping system may be linked to the dilution effect of carbon on N caused by improved water conditions and increased fertile spikes (Giuliani et al., 2011). Our study also implies the importance of grain weight per spike and N accumulation at maturity on simultaneous improvements in grain yield and protein content, rather than the NHI. This explanation is due to the fact that the NHI of modern wheat varieties is already near the highest value attributed to high mobility of N in plants (Kichey et al., 2007; Barraclough et al., 2010; Reynolds et al., 2012). Our results also confirmed the previous findings that fertile tillers affected root growth, N uptake, partitioning and utilization, resulting in variation of the equilibrium relationship between grain yield and protein content (Allard et al., 2013; Fernanda et al., 2013). Previous study has shown that only about 50% of N in ineffective tillers was remobilized in developing plants (Berry et al., 2003). Therefore, further improvements in N accumulation at maturity and grain protein content can be achieved by reducing ineffective tillers and selecting wheat varieties with high plant height, especially in the SW cropping system.

The grain protein quality of wheat is also heavily influenced by wet gluten content, gluten index, and their interactions (Williams et al., 2008). An improvement in the gluten index is of great importance for achieving high-yield and good-quality (Peng C. et al., 2022). In the present study, some wheat cultivars maintained a high gluten index without being influenced by changes in the environment, confirming that gluten index is mainly affected by genotype (Gil et al., 2011). Moreover, a significantly lower gluten index was recorded in MY-26 and CY-25, due to their high post anthesis N uptake. Previous studies attributed decreased gluten index resulting from increased post-anthesis N uptake to a decrease in N remobilization efficiency (Oosterom et al., 2010) and extension of the grain filling period (Gao et al., 2010; Hajas et al., 2018). In our study, the negative and positive correlations of post-anthesis N uptake with gluten index and 1,000-kernel weight, respectively, suggested that the decrease in gluten index resulted from simultaneous increases in post-anthesis N uptake and 1,000-kernel weight rather than post-anthesis N uptake alone. These results also supported the findings that both kernel weight (Slafer et al., 2014; Madry et al., 2015) and post- anthesis N uptake (Taulemesse et al., 2015; Worland et al., 2017) were determined by genotype. Therefore, we proposed a hypothesis that improved water conditions in the RW cropping system may decrease the gluten index of wheat varieties with high kernel weight by increasing their post-anthesis N uptake.

In addition, the interaction effects of environment and genotype on gluten index have also been reported (Mutwali et al., 2016; Sakr et al., 2021). Previous studies have demonstrated that the variation in gluten index was affected by soil water environments (Flagella et al., 2010). Soil water deficiency promotes the biosynthesis of storage proteins (Dong et al., 2012), dehydration (Shewry and Halford, 2002), and polymerizes glutenin to form glutenin macropolymer (Lerna et al., 2016), which will improve wheat protein quality (Ferreira et al., 2012). In the present study, higher N remobilization efficiency and gluten index in the SW cropping system in 2019–20 cropping year compared to 2018–19 cropping year were caused by a decrease in rainfall. Therefore, our results agreed with a finding that the improvement in gluten index can primarily be attributed to increased N remobilization efficiency under drought condition during grain filling stage (Moldestad et al., 2011). The novel findings of the present study were that the decreased N remobilization efficiency in relation to low grain number per spike in the SW cropping system was associated with the decrease in gluten index. Our study also confirmed that N remobilization in SW and RW cropping systems were regulated by source N supply and N demand of sink organs, respectively (Fischer, 2008; Serrago and Alzueta, 2013; Slimane et al., 2013). Future research will be focused on the onset of canopy and leaf senescence regulated by N remobilization, due to senescence which is the main factor that affects wheat grain yield and protein quality in the RW cropping system.

### Post-anthesis N uptake contributed to simultaneous improvements in grain yield and wet gluten content

In this study, the negative relationship between grain yield and grain protein content was observed in both wheat cropping systems, which was in agreement with previous results (Lollato et al., 2019; Nigro et al., 2019; Crosta et al., 2021). However, this negatively linear relationship in the RW cropping system was weaker than that in the SW cropping system, which confirmed previous findings that this negative correlation was affected by soil water and N environment (Dupont et al., 2006; Triboi et al., 2006). Genetic improvement in grain protein content was achieved at the expense of grain yield (Oury and Godin, 2007), which was consistent with our research results from the SW cropping system. Identifying wheat cultivars deviating from the negative relationship is essential to achieve both high grain yield and good protein quality. Previous studies have shown that genetic variation in grain protein deviation was linked to increases in N accumulation, N remobilization efficiency, and NHI (Jain et al., 2011; Suprayogi et al., 2011; Mosleth et al., 2015). In the present study, wheat cultivars with high plant height and late flowering date confer crops with high plant biomass and grain number per spike (Gaju et al., 2014), diluting the grain protein content. On the contrary, our research suggested that genetic selection for high post-anthesis N uptake and kernel weight contributed to increases in grain yield and wet gluten content by prolonging the photosynthetic duration and increasing the N accumulation at maturity (Bogard et al., 2010; Merah and Monneveux, 2015). Similar results were also observed elsewhere (Triboi and Triboi-Blondel, 2002; Brevis et al., 2010). Our results also confirmed these findings that the increased kernel weight could balance the decrease in grain number per unit area (Giunta et al., 2019), and the genetic variation in grain protein deviation was mainly determined by post-anthesis N uptake, rather than N remobilization efficiency (Mosleth et al., 2015). Furthermore, previous results have shown that post-anthesis N uptake was determined by genotype, and correlated positively with the root nitrate reductase gene (Zhao et al., 2013) and root system vigor (Nigro et al., 2019; Li et al., 2020; Lamichhane et al., 2021). Further analysis is required to analyze the contribution of N capture genes to grain protein deviation.

## Conclusion

In summary, genetic variation of grain yield in RW and SW cropping systems can be attributed to NUpE and NUtE, respectively. Fertile spikes in the RW cropping system and grain number per spike in the SW cropping system contributed to grain yield variation by 77.6 and 65.2%, respectively, due to variation in soil water and N availability. In both wheat cropping systems, the dwarf breeding strategy improved the NHI and HI of modern wheat, resulting in simultaneous improvements in grain yield and protein content. Improved water conditions in the RW cropping system increased post-anthesis N uptake, resulting in a decrease in the gluten index of wheat varieties with high kernel weight. Water deficit in the SW cropping system limited the N uptake and post-anthesis photosynthesis, resulting in a decreased contribution of fertile spikes and kernel weight to yield. Reducing ineffective tillers in the SW cropping system may be an effective strategy to increase grain number per spike and grain protein content maintaining high gluten index by improving the canopy structure and increasing the N accumulation of main stem and N remobilization efficiency. From these results, we concluded that plant height, kernel weight, and post-anthesis N uptake were the critically agronomic and NUE-related traits for the simultaneous selection of grain yield and protein quality. Our research results provided useful guidelines for improving both grain yield and protein quality by identifying desirable N-efficient genotypes in the two rotation systems.

## Data availability statement

The original contributions presented in the study are included in the article/supplementary material, further inquiries can be directed to the corresponding authors.

## Author contributions

YC: conceptualization, methodology, field management, data collection, data curation, and writing. KW: field management, data collection, and data curation. HC: field management and data collection. HY: conceptualization, methodology, writing review and editing, and supervision. TZ: conceptualization, methodology, writing review and editing, and supervision. XH: field management, data collection, and supervision. GF: funding acquisition, project administration, writing review and editing, and supervision. All authors contributed to the article and approved the submitted version.

## Funding

We acknowledge our laboratory members for help with field management and data collection. We are grateful for financial support from Sichuan Science and Technology Program (2022ZDZX0014), National Natural Science Foundation of China (32201904), National Key Research and Development Program of China (2016YFD0300406), Sichuan Science and Technology Program (2021YFYZ0002, 2021YJ0504), Agro-scientific Research in the Public Interest (20150312705) and Crops Breeding Project in Sichuan Province (2016NYZ0051).

## Conflict of interest

The authors declare that the research was conducted in the absence of any commercial or financial relationships that could be construed as a potential conflict of interest.

## Publisher's note

All claims expressed in this article are solely those of the authors and do not necessarily represent those of their affiliated organizations, or those of the publisher, the editors and the reviewers. Any product that may be evaluated in this article, or claim that may be made by its manufacturer, is not guaranteed or endorsed by the publisher.

## Abbreviations

Abbreviations: N: nitrogen
NUpE: N uptake efficiency
NUtE: N utilization efficiency
NUE: N use efficiency
NHI: N harvest index.
